# Satellite-Assisted Cell-Free Massive MIMO Systems with Multi-Group Multicast

**DOI:** 10.3390/s21186222

**Published:** 2021-09-16

**Authors:** Jiamin Li, Lingling Chen, Pengcheng Zhu, Dongming Wang, Xiaohu You

**Affiliations:** 1 National Mobile Communications Research Laboratory, Southeast University, Nanjing 210096, China; lijiamin@seu.edu.cn (J.L.); wangdm@seu.edu.cn (D.W.); xhyu@seu.edu.cn (X.Y.); 2 School of Information Science and Engineering, Southeast University, Nanjing 210096, China; 220190948@seu.edu.cn

**Keywords:** satellite-assisted, cell-free massive MIMO, multi-group multicast

## Abstract

In this paper, we use satellite-assisted and multi-group multicast mechanisms to relieve ground traffic pressure and improve data transmission efficiency of cell-free massive MIMO systems. We propose to estimate channel state information (CSI) by common pilot scheme. Given the estimated CSI, we derive the closed-form expressions of achievable rate with maximum ratio transmission (MRT) and zero-forcing (ZF) precoding. The correctness of the closed-form expressions is verified through simulations. The results show that with the help of satellite and multicast, the average system spectrum efficiency (SE) can be significantly improved.

## 1. Introduction

With the rapid development of mobile communication, more and more people and machines are connected to the network, and the exponential growth of data traffic has brought tremendous pressure to the terrestrial cellular network. This promotes the integration of the satellite network and terrestrial cellular network. Using satellites to assist terrestrial base stations, the data traffic of the ground cellular networks is shunted to provide users with better services. Moreover, satellite network can cope with the situation where limited local resources affect service quality by virtue of its global awareness. Therefore, the integration of satellite network and terrestrial network can effectively relieve ground traffic pressure and promote global information dissemination [1,2].

Recently, the concept of cell-free massive multiple-input multiple-output (MIMO) has attracted widespread attention in the academic community [3,4,5]. In cell-free massive MIMO systems, multiple access points (APs) jointly serve users within the same time-frequency resource, without existing cell. Ideally, all APs are connected to a central processing unit (CPU) to exchange information. The estimated channel state information (CSI) obtained by uplink pilot is used to precode the transmitted data in the downlink and perform data detection in the uplink. Moreover, the concept of cell-free massive MIMO is to combine massive MIMO [6] with distributed MIMO [7]. Therefore, it has the advantages of both massive MIMO and distributed MIMO [8].

Faced with the content-centric form of communication, hot information needs to be transmitted to multiple users, resulting that repeated transmission of information will bring a waste of resources [9]. Point-to-multipoint transmission using multicast can provide services to more users under the same resource conditions, therefore improving efficiency. In multicast scenario, users needing the same information are grouped into the same group, then multiple independent data streams are transmitted to different groups. Each group is called a multicasting group. Through multicast transmission, we can use the CSI at the transmitter to optimize the transmission based on a desired performance metric [10]. Ref. [11] introduced mixed unicast and multicast transmission under centralized massive MIMO systems, and performed multi-objective optimization to improve system performance. In [12], a non-orthogonal transmission framework based on layered-division multiplexing (LDM) is proposed to support both multicast and unicast services in cooperative multi-cell cellular networks. The above articles are all aimed at traditional centralized massive MIMO multicast systems. Considering the scenario of multi-group multicast cell-free massive MIMO, ref. [13] compared and analyzed the spectral efficiency (SE) after channel estimation and it is found that the use of downlink pilot can enhance the user’s ability to detect signals. A conjugate beamforming precoder was used in [14] to analyze the performance of multi-group multicast cell-free massive MIMO systems. They all consider only terrestrial cell-free massive MIMO systems with estimated CSI, not taking satellite-assisted into consideration.

Due to the growth of data traffic and the need to expand signal coverage, there have been many studies on the performance of integrated satellite ground multicast systems in recent years. Ref. [15] studied user access and resource allocation in the integrated satellite and terrestrial multi-cell multicast scenario, and formulated an optimal resource schedule scheme. In [16], a time-division multi-group multicast transmission scheme was proposed in the scenario of integrating satellites and terrestrial multi-cells, dividing the signal transmission into ground transmission phase and satellite transmission phase, which effectively improved system SE. The above research on satellite communication is based on ideal CSI which is not practical and will inevitably affect the performance of the system. Therefore, in this article, we will consider the non-ideal CSI and reasonably analyze the SE of the satellite-assisted terrestrial multicast systems.

In this paper, we consider satellite-assisted cell-free massive MIMO multi-group multicast scenarios. The users who need the same information are divided into the same group. APs and satellite multiplex the same bandwidth to provide multicast services for each group of users on the ground, and they are all equipped with multiple antennas. Assuming users in each multicast group receive the signals sent from the satellite and APs at the same time, then we use the estimated CSI to analyze the SE of the system. The main contributions of this paper are summarized as follows:

- We consider a multi-group multicast scheme in satellite-assisted cell-free massive MIMO systems. We use common pilot scheme to estimate satellite and terrestrial channel.
- With the estimated CSI, the closed-form expressions of the SE of multicast users with maximum ratio transmission (MRT) and zero-forcing (ZF) precoding schemes in satellite-assisted cell-free massive MIMO are derived. The SE of satellite-assisted cell-free massive MIMO multicast systems is analyzed.
- The accuracy of the derived closed-form expressions and the effectiveness of the multi-group multicast transmission scheme in satellite-assisted cell-free massive MIMO systems are verified. Insightful conclusion can be drawn that satellite-assisted can relieve terrestrial pressure and multicast can improve system SE effectively.

The remainder of the paper is organized as follows. In Section 2, we describe the system model including system configuration, satellite channel model, terrestrial channel model and signal transmission model. Section 3 consider the non-ideal CSI, using minimum mean-square error (MMSE) method to estimate satellite channel and terrestrial channel and the closed-form expressions of the MRT and ZF downlink achievable rate are derived. Representative numerical results are given in Section 4 before we conclude the paper in Section 5.

The following notations are used. All boldface letters stand for vectors (lower case) or matrices (upper case). $I_{N}$ is the size-*N* identity matrix. Italic letters (e.g., *X* or *x*) denote scalars. The transpose, Hermitian transpose and trace operators are denoted by ${\left(\cdot \right)}^{T}$, ${\left(\cdot \right)}^{H}$ and $\mathrm{tr}\left(\cdot \right)$, respectively. $C^{m\times n}$ denotes the set of m×n complex valued matrices. |x| is the absolute value of a scalar *x*, ∥X∥ is the spectral norm of a matrix X. $x∼\mathrm{CN}(0,\sigma^{2})$ means that *x* is a circularly symmetric complex Gaussian random variable with mean zero and variance $\sigma^{2}$. $E\left[\cdot \right]$ denotes the expectation operator.

## 2. System Model

### 2.1. System Configuration

**Figure 1:**
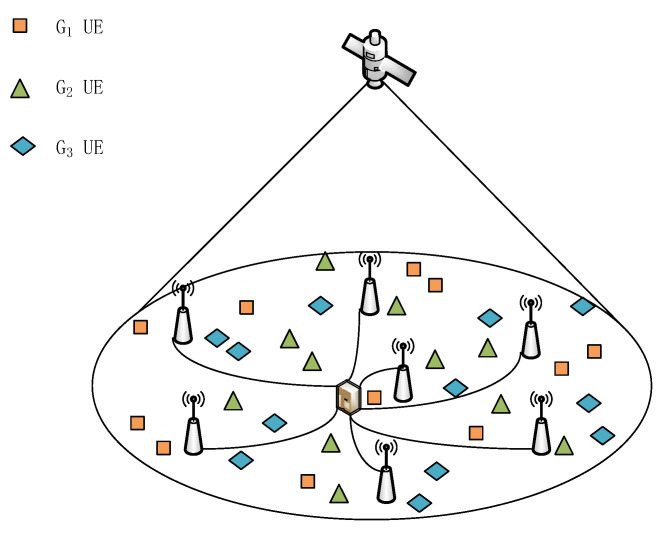
System Configuration.

We consider a satellite-assisted cell-free massive MIMO system where $N_{B}$ APs and a LEO multi-beam satellite provide multicast services to *K* single-antenna users in a cooperative manner. Assuming that the satellite and all APs use the same frequency spectrum during the entire transmission process, and all APs are distributed within the coverage area of the satellite. Each AP has $M_{B}$ antennas and the satellite has $M_{S}$ antennas. All users are grouped into *G* groups according to the requested contents. In multicast group *g*, the number of users is $K_{g}$. These users are arbitrarily distributed in the system. As shown in Figure 1, there are 34 users in total, which are divided into 3 multicast groups, then, $G=3,K_{1}=11,K_{2}=11,K_{3}=12$.

### 2.2. Channel Model

For the satellite channel, we assume that the satellite link is quasi-static. We do not take the influence of rain fading into consideration. The channel vector between the satellite and the *k*-th user in the *g*-th group is [17]

(1) $$g_{g,k}=L_{g,k}^{\frac{1}{2}}b_{g,k},\;g=1,2,\cdots ,G;\;k=1,2,\cdots ,K_{g},$$

where $L_{g,k}$ is large-scale attenuation and can be defined by

(2) $$L_{g,k}=\frac{G_{t}G_{r}B_{g,k}}{PL_{g,k}},$$

$G_{t}$ and $G_{r}$ represent satellite and user antenna gain, respectively. $PL_{g,k}$ is free space loss in dB [17],

(3) $$PL_{g,k}=90.45+20\mathrm{lg}\left(f_{c}\right)+20\mathrm{lg}\left(d_{g,k}\right),$$

$f_{c}$ is satellite carrier frequency, $d_{g,k}$ is the direct distance between the satellite and the user on the ground, $A_{g,k}$ is beam gain, $B_{g,k}={\left|\frac{J_{1}\left(u_{g,k}\right)}{2u_{g,k}}+36\frac{J_{3}\left(u_{g,k}\right)}{u_{g,k}^{3}}\right|}^{2}$, where $u_{g,k}=2.07123\frac{\sin\varphi_{g,k}}{\sin\varphi_{3dB}}$, $\varphi_{g,k}$ indicates the angle between the *k*-th user in the *g* group and the center of the corresponding beam, $\varphi_{3dB}$ represents the angle corresponding to the 3dB power loss of each spot beam, $J_{1}$ and $J_{3}$ are the first-order and third-order Bessel functions of the first kind, respectively. $b_{g,k}={\left[b_{g,k,1},b_{g,k,2},\cdots ,b_{g,k,M_{S}}\right]}^{T}$ is the small-scale attenuation between the satellite and the user, using the Shadowed Rice (SR) model [18], $b_{g,k,i}=A_{g,k,i}e^{j\psi_{g,k,i}}+Z_{g,k,i}e^{j\phi_{g,k,i}}$, where A and Z are the amplitudes of the scattering and line of sight (LoS) parts respectively, which obey the Rayleigh distribution and Nakagami-m distribution respectively. Assuming that ψ is a static random phase, uniformly distributed in $\left[0,2\pi \right)$, ϕ is the determined phase of the LoS part, which is independent of A and Z. Therefore, the SR distribution finally can be determined by the three parameters $\left(b,m,\Omega \right)$. The parameter values corresponding to different shadow levels can be referred to [18]. $\Omega =E\left[Z^{2}\right]$ indicates the average power of the LoS part, $2b=E\left[Z^{2}\right]$ is the average power of the scattering part. $m=E{\left[Z^{2}\right]}^{2}/\mathrm{Var}\left\{Z^{2}\right\}\geq 0$ represents the Nakagami parameter determined according to the severity of the attenuation. Then, the probability density function of ${\left|b_{g,k,i}\right|}^{2}$ can be expressed as

(4) $$f_{{\left|b_{g,k,i}\right|}^{2}}\left(x\right)={\left(\frac{2bm}{2bm+\Omega }\right)}^{m}\frac{x}{b}e^{\left(-\frac{x^{2}}{2b}\right)}B\left(b,m,\Omega ,x\right),$$

where x≥0, $B\left(b,m,\Omega ,x\right)={}_{1}F_{1}\left(m,1,\frac{\Omega x^{2}}{2b\left(2bm+\Omega \right)}\right)$ is Kummer function. Let $\eta_{g,k}$ be the statistical character of satellite channel.

It is worth noting that the channel model we consider here is a little different from the actual satellite channel. First, the satellite channel is actually constantly changing, and the influence of rain attenuation is also difficult to avoid, especially in the Ka-band. It becomes difficult for us to obtain real-time channel state information. Additionally, it may be that the currently acquired and estimated channel state information is no longer the current channel state. The channel aging problem also needs to be solved urgently. Secondly, if low-orbit satellites are considered, the Doppler frequency shift caused by the high-speed movement of the satellites actually needs to be taken into account in the channel model. Doppler frequency shift not only causes inter-symbol interference between signals. Doppler frequency shift spreading will also cause rapid time change of the channel, making it difficult for the receiving terminal to obtain instantaneous channel state information. Therefore, the estimation range of the Doppler frequency offset and the rate of change of the Doppler frequency offset during the integration process of the satellite system and the terrestrial 5G system need to be emphatically considered and urgently needed to be resolved.

For the terrestrial channel, we apply uncorrelated Rayleigh fading channel. The channel vector between APs and the *k*-th user in the *g* group can be expressed as

(5) $$h_{g,k}=\beta_{g,k}^{\frac{1}{2}}u_{g,k},\;g=1,2,\cdots ,G;\;k=1,2,\cdots ,K_{g},$$

where $\beta_{g,k}=\mathrm{diag}\left(\beta_{1,g,k},\beta_{2,g,k},\cdots ,\beta_{N_{B},g,k}\right)⊗I_{M_{B}}$, $\beta_{n,g,k}=d_{n,g,k}^{-a}$ represents the large-scale attenuation between the *n*-th AP and the *k*-th user in group *g*, *a* is the path loss factor, generally between 3.0 and 5.0, $d_{n,g,k}$ represents the distance between the *n*-th AP and the *k*-th user in group *g*, $u_{g,k}$ is uncorrelated small-scale fast fading vector, $u_{g,k}∼\mathrm{CN}\left(0,I_{N_{B}M_{B}}\right)$, including independent identically distributed cyclic symmetric complex Gaussian (ZMCSCG) random variables with a mean of 0 and a variance of 1.

## 3. Performance Analysis

### 3.1. Channel Estimation

Obtaining accurate channel state information is particularly important in wireless communication transmission. In an actual communication scenario, neither the transmitter nor the receiver may be able to fully understand the channel state. At this time, it is necessary to estimate the channel state information. The channel state information on the receiver side can be obtained through pilot transmission. The channel state information on the transmitter side can be obtained through the feedback link or channel reciprocity. According to [19], the estimation accuracy of the channel state information is inconsistent, resulting in inconsistent system capacity. It can be seen that when the estimation error increases, the system capacity decreases. It can be seen that accurate estimation of channel state information is particularly important.

In this paper, we use pilot-assisted channel estimation. Due to the channel reciprocity, the CSI obtained by uplink channel estimation can be used for the precoding of downlink data transmission. In multicast transmission, each multicast group has many users. Take a TV channel for multicast transmission as an example, when hundreds of users watch the same channel, the resources in the coherent range will be exhausted. Therefore, for multicast users, it is more appropriate to adopt the new common pilot allocation scheme mentioned in [20]. Users in each multicast group share the same pilot, so a total of *G* pilots are required instead of $\sum_{g=1}^{G}K_{g}$ pilots. The length of pilot sequence is $\tau_{p}$.

For the satellite channel, the pilot sequence transmitted by the *g*-th user group is $\psi_{g}$, $\psi_{g}\in C^{\tau_{p}\times 1}$, and the pilot signal received by the satellite is expressed as

(6) $$r_{p}=\sqrt{\tau_{p}\rho_{p,S}}\sum_{g=1}^{G}\sum_{k=1}^{K_{g}}g_{g,k}\psi_{g}^{H}+N_{p,S}.$$

The MMSE estimate of the satellite channel is [21]

(7) $${\hat{g}}_{g,k}=\frac{\sqrt{\tau_{p}\rho_{p,S}}\eta_{g,k}}{\tau_{p}\rho_{p,S}\sum_{k^{\prime }=1}^{K_{g}}\eta_{g,k^{\prime }}+1}\left(\sqrt{\tau_{p}\rho_{p,S}}\sum_{k=1}^{K_{g}}g_{g,k}+n_{S,g}\right)=\gamma_{g,k}^{1/2}{\hat{b}}_{g,k},$$

where $n_{S,g}∼\mathrm{CN}\left(0,I_{M_{S}}\right)$ is standardized additive noise, $\rho_{p,S}$ is the power of uplink pilot, $\gamma_{g,k}=\frac{\tau_{p}\rho_{p,S}\eta_{g,k}^{2}}{\tau_{p}\rho_{p,S}\sum_{k^{\prime }=1}^{K_{g}}\eta_{g,k^{\prime }}+1}$ is the equivalent large-scale fading and ${\hat{b}}_{g,k}∼\mathrm{CN}\left(0,I_{M_{S}}\right)$.

Let $g_{g}=\sum_{t=1}^{K_{g}}\sqrt{\tau_{p}\rho_{p,S}}g_{g,t}$ represent the linear superposition of the user channels used in group *g*, then the estimated value can be expressed as

(8) $${\hat{g}}_{g}=\frac{\sum_{t=1}^{K_{g}}\tau_{p}\rho_{p,S}\eta_{g,t}}{\tau_{p}\rho_{p,S}\sum_{k^{\prime }=1}^{K_{g}}\eta_{g,k^{\prime }}+1}\left(\sqrt{\tau_{p}\rho_{p,S}}\sum_{k=1}^{K_{g}}g_{g,k}+n_{S,g}\right)=\varepsilon_{g}^{1/2}{\hat{b}}_{g},$$

where $\varepsilon_{g}=\frac{{\left(\sum_{t=1}^{K_{g}}\tau_{p}\rho_{p,S}\eta_{g,t}\right)}^{2}}{\tau_{p}\rho_{p,S}\sum_{k^{\prime }=1}^{K_{g}}\eta_{g,k^{\prime }}+1}$ is the equivalent large-scale fading and ${\hat{b}}_{g}∼\mathrm{CN}\left(0,I_{M_{S}}\right)$.

Using a $M_{S}\times G$ matrix $\hat{G}=\left[{\hat{g}}_{1},{\hat{g}}_{2},\cdots ,{\hat{g}}_{G}\right]$ to represent the channel estimation vector between the satellite and users in all G multicast groups, as a result, there is only one scalar coefficient between ${\hat{g}}_{g,k}$ and ${\hat{g}}_{g}$,

(9) $$\begin{array}{cc} {\hat{g}}_{g,k} & =c_{g,k}{\hat{g}}_{g} \end{array}$$

(10) $$\begin{array}{cc} c_{g,k} & =\frac{\sqrt{\tau_{p}\rho_{p,S}}\eta_{g,k}}{\sum_{t=1}^{K_{g}}\tau_{p}\rho_{p,S}\eta_{g,t}}. \end{array}$$

Similarly, the MMSE estimate of the terrestrial channel is

(11) $${\hat{h}}_{g,k}=\chi_{g,k}^{1/2}{\hat{u}}_{g,k},$$

where $\chi_{g,k}=\mathrm{diag}\left(\chi_{1,g,k},\chi_{2,g,k},\cdots ,\chi_{N_{B},g,k}\right)⊗I_{M_{B}}$ is the equivalent large-scale fading, $\chi_{n,g,k}=\frac{\tau_{p}\rho_{p,B}\beta_{n,g,k}}{\tau_{p}\rho_{p,B}\sum_{k^{\prime }=1}^{K_{g}}\beta_{n,g,k^{\prime }}+1}$ and ${\hat{u}}_{g,k}∼\mathrm{CN}\left(0,I_{N_{B}M_{B}}\right)$.

The estimated value of $h_{g}$ can be expressed as

(12) $${\hat{h}}_{g}=\alpha_{g}^{1/2}{\hat{u}}_{g},$$

where $\alpha_{g}=\mathrm{diag}\left(\alpha_{1,g},\alpha_{2,g},\cdots ,\alpha_{N_{B},g}\right)⊗I_{M_{B}}$ is the equivalent large-scale fading, $\alpha_{n,g}=\frac{{\left(\sum_{t=1}^{K_{g}}\tau_{p}\rho_{p,B}\beta_{n,g,t}\right)}^{2}}{\sum_{k^{\prime }=1}^{K_{g}}\tau_{p}\rho_{p,B}\beta_{n,g,k^{\prime }}+1}$ and ${\hat{u}}_{g}∼\mathrm{CN}\left(0,I_{N_{B}M_{B}}\right)$. Using a $N_{B}M_{B}\times G$ matrix $\hat{H}=\left[{\hat{h}}_{1},{\hat{h}}_{2},\cdots ,{\hat{h}}_{G}\right]$ to represent the channel estimation vector between APs and users in all G multicast groups, as a result, there is a diagonal matrix difference between ${\hat{h}}_{g,k}$ and ${\hat{h}}_{g}$,

(13) $${\hat{h}}_{g,k}=\kappa_{g,k}{\hat{h}}_{g},$$

where $\kappa_{g,k}=\mathrm{diag}\left(\kappa_{1,g,k},\kappa_{2,g,k},\cdots ,\kappa_{N_{B},g,k}\right)⊗I_{M_{B}}$ and $\kappa_{n,g,k}=\frac{\sqrt{\tau_{p}\rho_{p,B}}\beta_{n,g,k}}{\sum_{t=1}^{K_{g}}\tau_{p}\rho_{p,B}\beta_{n,g,t}}$.

### 3.2. Downlink Spectral Efficiency Analysis

In satellite-assisted cell-free massive MIMO multi-group multicast systems, APs and the satellite provide multicast services for *G* group users. We assume that users in each multicast group receive the signals sent from the satellite and APs at the same time [16]. For downlink signal transmission, the multicast signal received by the *k*-th user in group *g* is expressed as

(14) $$y_{g,k}=h_{g,k}^{H}w_{g}s_{g}+g_{g,k}^{H}v_{g}s_{g}+h_{g,k}^{H}\sum_{i=1,i\neq g}^{G}w_{i}s_{i}+g_{g,k}^{H}\sum_{i=1,i\neq g}^{G}v_{i}s_{i}+n_{g,k},$$

where $s_{g}\in C$ indicates the data symbol sent to users in group *g*, and $E\left[{\left|s_{g}\right|}^{2}\right]=1$, $n_{g,k}∼\mathrm{CN}\left(0,\sigma_{dl}^{2}\right)$ is additive noise, $w_{g}\in C^{N_{B}M_{B}\times 1}$ and $v_{g}\in C^{M_{S}\times 1}$ represent the precoding vectors sent by APs and satellite to group *g*, respectively.

It is worth noting that we do not take the interference between satellite systems and terrestrial systems into consideration. However, during the integration process of satellite systems and terrestrial systems, the inter-system interference caused by frequency sharing is also worth considering.

Assuming that users only use statistical channel state information for signal decoding, according to [22], the received signal can be rewritten as

(15) $$y_{g,k}=E\left[h_{g,k}^{H}w_{g}+g_{g,k}^{H}v_{g}\right]+n_{g,k}^{\prime },$$

where

(16) $$n_{g,k}^{\prime }=\left[h_{g,k}^{H}w_{g}+g_{g,k}^{H}v_{g}-E\left[h_{g,k}^{H}w_{g}+g_{g,k}^{H}v_{g}\right]\right]s_{g}+h_{g,k}^{H}\sum_{i=1,i\neq g}^{G}w_{i}s_{i}+g_{g,k}^{H}\sum_{i=1,i\neq g}^{G}v_{i}s_{i}+n_{g,k}.$$

$E\left[h_{g,k}^{H}w_{g}+g_{g,k}^{H}v_{g}\right]s_{g}$ is the only expected signal of the *k*-th user in group *g*, $n_{g.k}^{\prime }$ is interference, then the achievable SE of the *k*-th user in group *g* can be expressed as

(17) $$R_{g,k}=\log_{2}\left(1+\frac{{\left|E\left[h_{g,k}^{H}w_{g}+g_{g,k}^{H}v_{g}\right]\right|}^{2}}{\sum_{i=1}^{G}E\left[{\left|h_{g,k}^{H}w_{i}+g_{g,k}^{H}v_{i}\right|}^{2}\right]-{\left|E\left[h_{g,k}^{H}w_{g}+g_{g,k}^{H}v_{g}\right]\right|}^{2}+\sigma_{dl}^{2}}\right).$$

In this paper, we consider MRT and ZF precoding. For MRT precoding, the precoding vector of the terrestrial base station for the *g*-th multicast group is

(18) $$w_{g}^{\mathrm{mrt}}=p_{B,g}^{\mathrm{dl}}{\hat{h}}_{g},$$

where $p_{B,g}^{\mathrm{dl}}=1/\sqrt{E[∥{\hat{h}}_{g}{∥}^{2}]}=1/\sqrt{M_{B}\sum_{m=1}^{N_{B}}\alpha_{m,g}}$ is power normalization parameter, and $E\left[∥w_{g}^{\mathrm{mrt}}{∥}^{2}\right]=1$. The precoding vector of the satellite for the *g*-th multicast group is

(19) $$v_{g}^{\mathrm{mrt}}=p_{S,g}^{\mathrm{dl}}{\hat{g}}_{g},$$

where $p_{S,g}^{\mathrm{dl}}=1/\sqrt{E[∥{\hat{g}}_{g}{∥}^{2}]}=1/\sqrt{M_{S}\varepsilon_{g}}$ is power normalization parameter, and $E\left[∥v_{g}^{\mathrm{mrt}}{∥}^{2}\right]=1$.

**Theorem** **1.** *When the base station and satellite terminals use MRT precoding, the closed-form expression of the downlink achievable rate can be given by $R_{g,k}^{\mathrm{mrt}}=\log_{2}\left(1+SINR_{g,k}^{mrt}\right)$*

(20) $$\;SINR_{g,k}^{mrt}=\frac{{\left(p_{B,g}^{\mathrm{dl}}M_{B}\sum_{m=1}^{N_{B}}\kappa_{m,g,k}\alpha_{m,g}+p_{S,g}^{\mathrm{dl}}M_{S}\varepsilon_{g}c_{g,k}\right)}^{2}}{\sum_{i=1}^{G}\left[\sum_{m=1}^{N_{B}}\left(\beta_{m,g,k}-\chi_{m,g,k}\right)+\eta_{g,k}-\gamma_{g,k}\right]-2p_{B,g}^{\mathrm{dl}}p_{S,g}^{\mathrm{dl}}M_{B}M_{S}\varepsilon_{g}c_{g,k}\sum_{m=1}^{N_{B}}\kappa_{m,g,k}\alpha_{m,g}+\sigma_{dl}^{2}}.$$

**Proof** **of** **Theorem 1.** The proof is given in Appendix A. □

For ZF precoding, the precoding vector of the terrestrial base station for the *g*-th multicast group is

(21) $$w_{g}^{\mathrm{ZF}}=q_{B,g}^{\mathrm{dl}}a_{B,g},$$

where $a_{B,g}$ is the *g*-th column of $\hat{H}{\left({\hat{H}}^{H}\hat{H}\right)}^{-1}$, $q_{B,g}^{\mathrm{dl}}=1/\sqrt{E[∥a_{B,g}{∥}^{2}]}=1/\sqrt{\left(M_{B}-G\right)\sum_{m=1}^{N_{B}}\alpha_{m,g}}$ is power normalization parameter, and $E\left[∥w_{g}^{\mathrm{ZF}}{∥}^{2}\right]=1$. The precoding vector of the satellite for the *g*-th multicast group is

(22) $$v_{g}^{\mathrm{ZF}}=q_{S,g}^{\mathrm{dl}}a_{S,g},$$

where $a_{S,g}$ is the *g*-th column of $\hat{G}{\left({\hat{G}}^{H}\hat{G}\right)}^{-1}$, $q_{S,g}^{\mathrm{dl}}=1/\sqrt{E[∥a_{S,g}{∥}^{2}]}=1/\sqrt{\left(M_{S}-G\right)\varepsilon_{g}}$ is power normalization parameter, and $E\left[∥v_{g}^{\mathrm{ZF}}{∥}^{2}\right]=1$.

**Theorem** **2.** *When the base station and satellite terminals use ZF precoding, the closed expression of the downlink achievable rate can be given by $R_{g,k}^{\mathrm{ZF}}=\log_{2}\left(1+SINR_{g,k}^{ZF}\right)$*

(23) $$\;SINR_{g,k}^{ZF}=\frac{{\left(q_{B,g}^{\mathrm{dl}}\sum_{m=1}^{N_{B}}\kappa_{m,g,k}+q_{S,g}^{\mathrm{dl}}c_{g,k}\right)}^{2}}{\sum_{i=1}^{G}\sum_{m=1}^{N_{B}}\left(\beta_{m,g,k}-\chi_{m,g,k}\right)+\sum_{i=1}^{G}\left(\eta_{g,k}-\gamma_{g,k}\right)-2q_{B,g}^{\mathrm{dl}}q_{S,g}^{\mathrm{dl}}c_{g,k}\sum_{m=1}^{N_{B}}\kappa_{m,g,k}+\sigma_{dl}^{2}}.$$

**Proof** **of** **Theorem 2.** The proof is given in Appendix B. □

**Remark** **1.** *In Theorems 1 and 2, we derive the closed-form expressions of achievable rate with MRT and ZF precoding. In contrast to the derivation process under only terrestrial multicast scenario, satellite-assisted multicast introduces an additional item $g_{g,k}^{H}v_{g}$. It increases the difficulty of derivation. By using large-scale random matrix theory and introducing middle term between terrestrial item and satellite item, we successfully derive the closed-form expressions.*

**Remark** **2.** *The closed-form expressions of achievable rate with MRT and ZF precoding presented in Theorems 1 and 2 provide convenience for parameter analyses. Compared with simulation expression, there might be a little different. However, it is relatively tractable and accurate. In particular, with closed-form expressions, there is no need to characterize the distributions of signal and interference powers to obtain an analytical expression, so that we can simplify the analysis process, but achieve relatively accurate results simultaneously.*

## 4. Numerical Results

In this section, the theoretical analysis presented in Section 3 is verified through a set of Monte Carlo simulations, to analyze the performance of the satellite-assisted cell-free massive MIMO multi-group multicast system.

### 4.1. Simulation Parameter Setting

In the simulation, we consider a circular cell-free massive MIMO system, where all APs and users are randomly distributed and the satellite cover the entire system, so that the satellite and terrestrial base stations provide users with multicast services cooperatively. All the simulation parameters are shown in Table 1.

### 4.2. Simulation Result Analysis

**Figure 2:**
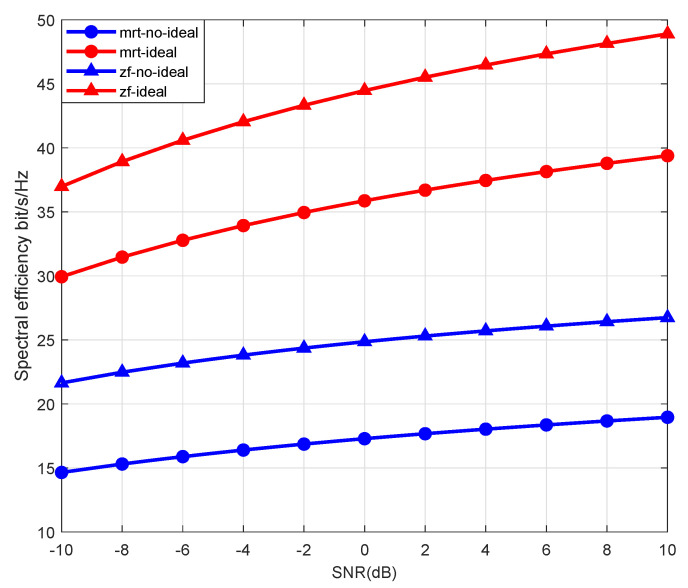
SE against SNR with MRT and ZF beamforming under ideal and non-ideal CSI.

SE is shown in Figure 2 against signal-noise ratio (SNR) with MRT and ZF precoding under ideal and non-ideal CSI. In addition, the number of AP antennas and satellite antennas is set as 60 and 30, respectively. It can be seen that the performance of ZF precoding is better than MRT precoding. Simultaneously, the SE of the system under ideal CSI is significantly higher than that under non-ideal channels. This also reflects the importance of obtaining accurate channel state information.

**Figure 3:**
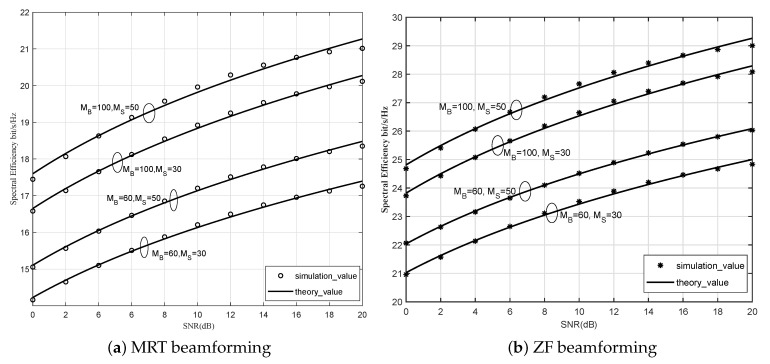
SE against SNR with MRT and ZF beamforming.

SE is shown in Figure 3 against SNR with different number of antennas at APs and satellite. It can be seen that the theoretical results agree well with the simulated results, which verifies the accuracy of the closed-form expressions derived in Theorems 1 and 2. As the number of satellite antennas increases, the SE increases, and the performance of ZF precoding is better than MRT precoding.

Please note that the coexistence of satellite and terrestrial networks may introduce complicated interference problems. When the terrestrial network and the satellite network transmit signals to users at the same time, the repeated use of the same frequency causes inter-system interference which may degrade the system performance severely. How to decrease the inter-system interference will be considered to be a point for further research.

**Figure 4:**
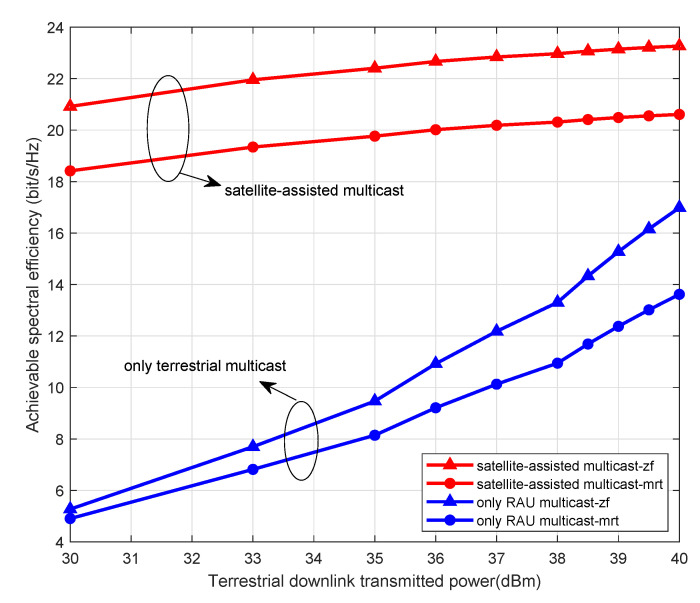
SE against downlink transmitted power under satellite-assisted multicast and only terrestrial multicast.

Figure 4 illustrates the average SE against terrestrial downlink transmit power under two scenarios of satellite-assisted multicast and only terrestrial unicast. We set SNR as 10 dB. Meanwhile, we assume that the terrestrial downlink transmitted power of ranging from 30 dBm to 40 dBm and the satellite downlink transmitted power as 30 dBm. Simultaneously, the number of AP antennas and satellite antennas is set as 60 and 30, respectively. It can be seen in Figure 4 that the SE increases with the increasing terrestrial downlink transmitted power. In addition, the system we are studying is satellite-assisted cell-free massive MIMO systems, including satellite networks and terrestrial networks. The satellite network serves as a supplement to the relatively mature terrestrial network. Therefore, when the terrestrial network traffic is overloaded, part of the traffic is offloaded to the satellite network, therefore relieving the communication pressure on the ground. It is reflected in Figure 4. With the help of satellite, when the user’s total rate is constant, part of the rate of ground base station is offloaded to the satellite, resulting that the rate in only terrestrial multicast scene is reduced.

**Figure 5:**
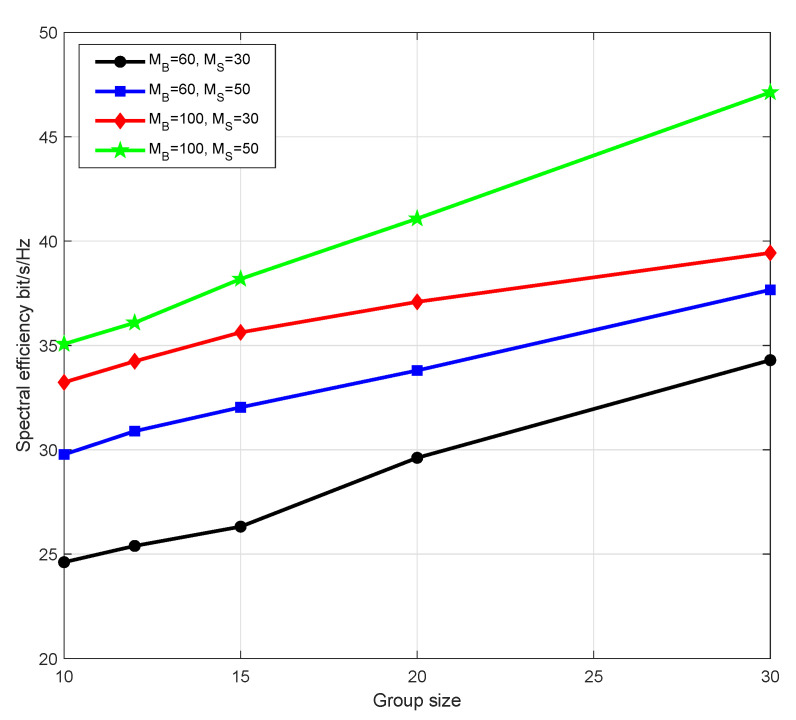
System spectral efficiency of different group size.

Figure 5 illustrates the influence of different group size on the system SE. The total number of users is set to 60. We change the number of groups, and thus the number of users in each group has also changed. The horizontal coordinate in Figure 5 is the number of users in each group, $\left\{10,12,15,20,30\right\}$ correspondingly. Multicast provides a one-time data transmission service for multiple users who need the same resources. It not only avoids repeated transmission of data to improve transmission efficiency, but also ensures that it does not affect other users needing different information to receive data effectively. This can be reflected in Figure 5 that the system SE increases with group size, indicating that multicast scheme helps improve the system performance.

## 5. Conclusions

In this paper, we analyzed the downlink spectral efficiency of a satellite-assisted cell-free massive MIMO multi-group multicast system, trying to merge the satellite system with the terrestrial cell-free system. First, we use the SR model and the Rayleigh model to establish the satellite channel and the ground channel, and use the common pilot scheme to estimate the satellite and ground CSI. Then, given the estimated CSI, we derived the closed-form expressions of the downlink achievable rate with MRT and ZF beamforming. Finally, the correctness of the derived closed-form expressions was verified, and the simulation results revealed that with the help of multicast, higher system SE can be obtained. Moreover, the integration of satellite and terrestrial network effectively relieved the pressure of terrestrial communication. In the future, we will focus on the study of how to decrease the inter-system interference caused by the coexistence of satellite and terrestrial networks.

## Figures and Tables

**Table 1:** Simulation parameters.

Parameter Name	Value
Orbit altitude $d_{0}$ 1350 km	
Carrier frequency $f_{c}$	20 GHz
The number of AP $N_{B}$	6
The number of multicast group *G*	3
The number of users per group $K_{g}$	5
Uplink pilot signal transmit power $\rho_{p}$	30 dBm
Cell radius *r*	1 km
Path loss index *a*	3.7
Satellite transmitting antenna gain $G_{t}$	24.3 dBi
User receiving antenna gain $G_{r}$	10 dBi
3 dB angle $\varphi_{3dB}$	$0.4^{\circ }$

## Data Availability

Not applicable.

## Appendix A. Proof of Theorem 1

To obtain the closed-form expression of the downlink achievable rate with MRT beamforming, the following four items need to be calculated: $E\left[h_{g,k}^{H}w_{g}^{\mathrm{mrt}}\right]$, $E\left[g_{g,k}^{H}v_{g}^{\mathrm{mrt}}\right]$, $\sum_{i=1}^{G}E\left[|h_{g,k}^{H}w_{i}^{\mathrm{mrt}}{|}^{2}\right]$ and $\sum_{i=1}^{G}E\left[|g_{g,k}^{H}v_{i}^{\mathrm{mrt}}{|}^{2}\right]$.

For the term $E\left[h_{g,k}^{H}w_{g}^{\mathrm{mrt}}\right]$, we have 2.0

(A1) $$\begin{array}{cc} E\left[h_{g,k}^{H}w_{g}^{\mathrm{mrt}}\right] & =E\left[p_{B,g}^{\mathrm{dl}}h_{g,k}^{H}{\hat{h}}_{g}\right]\\ \; & \overset{\left(a\right)}{=}E\left[p_{B,g}^{\mathrm{dl}}{\hat{h}}_{g,k}^{H}{\hat{h}}_{g}\right]\\ \; & \overset{\left(b\right)}{=}E\left[p_{B,g}^{\mathrm{dl}}{\hat{h}}_{g}^{H}\kappa_{g,k}^{H}{\hat{h}}_{g}\right]\\ \; & \overset{\left(c\right)}{=}p_{B,g}^{\mathrm{dl}}M_{B}\sum_{m=1}^{N_{B}}\kappa_{m,g,k}\alpha_{m,g}, \end{array}$$

where $\left(a\right)$ is because ${\tilde{h}}_{g,k}$ and ${\hat{h}}_{g,k}$ are independent, $\left(b\right)$ results from (13) and (c) are obtained by the large-scale random matrix conclusion Theorem 1 in [23].

Similarly, for the term $E\left[g_{g,k}^{H}v_{g}^{\mathrm{mrt}}\right]$, we have

(A2) $$\begin{array}{cc} E\left[g_{g,k}^{H}v_{g}^{\mathrm{mrt}}\right] & \overset{\left(a\right)}{=}E\left[p_{S,g}^{\mathrm{dl}}c_{g,k}{\hat{g}}_{g}^{H}{\hat{g}}_{g}\right]\\ \; & \overset{\left(b\right)}{=}p_{S,g}^{\mathrm{dl}}M_{S}\varepsilon_{g}c_{g,k}, \end{array}$$

For the term $\sum_{i=1}^{G}E\left[{\left|h_{g,k}^{H}w_{i}^{\mathrm{mrt}}\right|}^{2}\right]$, we have

(A3) $$\;\begin{array}{cc} \sum_{i=1}^{G}E\left[{\left|h_{g,k}^{H}w_{i}^{\mathrm{mrt}}\right|}^{2}\right] & =\sum_{i=1}^{G}E\left[|p_{B,i}^{\mathrm{dl}}h_{g,k}^{H}{\hat{h}}_{i}{|}^{2}\right]\\ \; & \overset{\left(a\right)}{=}\sum_{i=1}^{G}{\left(p_{B,i}^{\mathrm{dl}}\right)}^{2}E\left[|{\hat{h}}_{g}^{H}\kappa_{g,k}^{H}{\hat{h}}_{i}{|}^{2}+|{\tilde{h}}_{g,k}^{H}{\hat{h}}_{i}{|}^{2}\right]\\ \; & \overset{\left(b\right)}{=}{\left(p_{B,g}^{\mathrm{dl}}\right)}^{2}E\left[|{\hat{h}}_{g}^{H}\kappa_{g,k}^{H}{\hat{h}}_{g}{|}^{2}\right]+\sum_{i=1}^{G}{\left(p_{B,i}^{\mathrm{dl}}\right)}^{2}E\left[|{\tilde{h}}_{g,k}^{H}{\hat{h}}_{i}{|}^{2}\right]\\ \; & \overset{\left(c\right)}{=}{\left(p_{B,g}^{\mathrm{dl}}M_{B}\sum_{m=1}^{N_{B}}\kappa_{m,g,k}\alpha_{m,g}\right)}^{2}+\sum_{i=1}^{G}\sum_{m=1}^{N_{B}}\left(\beta_{m,g,k}-\chi_{m,g,k}\right), \end{array}$$

where $\left(a\right)$ results from the independence of ${\tilde{h}}_{g,k}$ and ${\hat{h}}_{g,k}$, and the nature of variance. $\left(b\right)$ is obtained because that ${\hat{h}}_{g}$ and ${\hat{h}}_{i}$ are uncorrelated random vectors without considering pilot pollution. (c) is obtained because of the large-scale random matrix conclusion.

Considering the term $\sum_{i=1}^{G}E\left[|g_{g,k}^{H}v_{i}^{\mathrm{mrt}}{|}^{2}\right]$, we have

(A4) $$\begin{array}{cc} \sum_{i=1}^{G}E\left[{\left|g_{g,k}^{H}v_{i}^{\mathrm{mrt}}\right|}^{2}\right] & =\sum_{i=1}^{G}E\left[{\left|p_{S,i}^{\mathrm{dl}}g_{g,k}^{H}{\hat{g}}_{i}\right|}^{2}\right]\\ \; & \overset{\left(a\right)}{=}\sum_{i=1}^{G}{\left(p_{S,i}^{\mathrm{dl}}\right)}^{2}E\left[|c_{g,k}{\hat{g}}_{g}^{H}{\hat{g}}_{i}{|}^{2}+|{\tilde{g}}_{g,k}^{H}{\hat{g}}_{i}{|}^{2}\right]\\ \; & \overset{\left(b\right)}{=}{\left(p_{S,g}^{\mathrm{dl}}\right)}^{2}E\left[|c_{g,k}{\hat{g}}_{g}^{H}{\hat{g}}_{g}{|}^{2}\right]+\sum_{i=1}^{G}{\left(p_{S,i}^{\mathrm{dl}}\right)}^{2}E\left[|{\tilde{g}}_{g,k}^{H}{\hat{g}}_{i}{|}^{2}\right]\\ \; & \overset{\left(c\right)}{=}{\left(p_{S,g}^{\mathrm{dl}}M_{S}\varepsilon_{g}c_{g,k}\right)}^{2}+\sum_{i=1}^{G}\left(\eta_{g,k}-\gamma_{g,k}\right), \end{array}$$

where $\left(a\right)$ results from the independence of ${\tilde{g}}_{g,k}$ and ${\hat{g}}_{g,k}$, and the nature of variance. $\left(b\right)$ is obtained because that ${\hat{g}}_{g}$ and ${\hat{g}}_{i}$ are uncorrelated random vectors without considering pilot pollution. (c) is obtained by the large-scale random matrix conclusion.

Substituting the above results into (17) yields the closed-form expression of the downlink achievable rate with MRT beamforming.

## Appendix B. Proof of ZF Closed-Form Expression

To obtain the closed-form expression of the downlink achievable rate with ZF beamforming, the following four items need to be calculated: $E\left[h_{g,k}^{H}w_{g}^{\mathrm{ZF}}\right]$, $E\left[g_{g,k}^{H}v_{g}^{\mathrm{ZF}}\right]$, $\sum_{i=1}^{G}E\left[|h_{g,k}^{H}w_{i}^{\mathrm{ZF}}{|}^{2}\right]$, $\sum_{i=1}^{G}E\left[|g_{g,k}^{H}v_{i}^{\mathrm{ZF}}{|}^{2}\right]$.

For the term $E\left[h_{g,k}^{H}w_{g}^{\mathrm{ZF}}\right]$, we have

(A5) $$\begin{array}{cc} E\left[h_{g,k}^{H}w_{g}^{\mathrm{ZF}}\right] & =E\left[q_{B,g}^{\mathrm{dl}}h_{g,k}^{H}\hat{H}{\left({\hat{H}}^{H}\hat{H}\right)}^{-1}e_{g}\right]\\ \; & \overset{\left(a\right)}{=}E\left[q_{B,g}^{\mathrm{dl}}{\hat{h}}_{g}^{H}\kappa_{g,k}^{H}\hat{H}{\left({\hat{H}}^{H}\hat{H}\right)}^{-1}e_{g}\right]\\ \; & \overset{\left(b\right)}{=}q_{B,g}^{\mathrm{dl}}\sum_{m=1}^{N_{B}}\kappa_{m,g,k}, \end{array}$$

where $\left(a\right)$ results from the independence of ${\tilde{h}}_{g,k}$ and ${\hat{h}}_{g,k}$ and (13), $\left(b\right)$ is obtained because $E\left[{\hat{h}}_{g}^{H}\hat{H}{\left({\hat{H}}^{H}\hat{H}\right)}^{-1}e_{g}\right]=1$.

Similarly, for the term $E\left[g_{g,k}^{H}v_{g}^{\mathrm{ZF}}\right]$, we have

(A6) $$\begin{array}{cc} E\left[g_{g,k}^{H}v_{g}^{\mathrm{ZF}}\right] & =E\left[q_{S,g}^{\mathrm{dl}}g_{g,k}^{H}\hat{G}{\left({\hat{G}}^{H}\hat{G}\right)}^{-1}e_{g}\right]\\ \; & \overset{\left(a\right)}{=}E\left[q_{S,g}^{\mathrm{dl}}c_{g,k}{\hat{g}}_{g}^{H}\hat{G}{\left({\hat{G}}^{H}\hat{G}\right)}^{-1}e_{g}\right]\\ \; & \overset{\left(b\right)}{=}q_{S,g}^{\mathrm{dl}}c_{g,k}, \end{array}$$

For the term $\sum_{i=1}^{G}E\left[|h_{g,k}^{H}w_{i}^{\mathrm{ZF}}{|}^{2}\right]$, we have

(A7) $$\;\begin{array}{cc} \sum_{i=1}^{G}E\left[{\left|h_{g,k}^{H}w_{i}^{\mathrm{ZF}}\right|}^{2}\right] & =\sum_{i=1}^{G}E\left[|q_{B,i}^{\mathrm{dl}}h_{g,k}^{H}\hat{H}{\left({\hat{H}}^{H}\hat{H}\right)}^{-1}e_{i}{|}^{2}\right]\\ \; & \overset{\left(a\right)}{=}\sum_{i=1}^{G}{\left(q_{B,i}^{\mathrm{dl}}\right)}^{2}E\left[|{\hat{h}}_{g}^{H}\kappa_{g,k}^{H}\hat{H}{\left({\hat{H}}^{H}\hat{H}\right)}^{-1}e_{i}{|}^{2}+|{\tilde{h}}_{g,k}^{H}\hat{H}{\left({\hat{H}}^{H}\hat{H}\right)}^{-1}e_{i}{|}^{2}\right]\\ \; & \overset{\left(b\right)}{=}{\left(q_{B,g}^{\mathrm{dl}}\right)}^{2}E\left[|{\hat{h}}_{g}^{H}\kappa_{g,k}^{H}\hat{H}{\left({\hat{H}}^{H}\hat{H}\right)}^{-1}e_{g}{|}^{2}\right]+\sum_{i=1}^{G}{\left(q_{B,i}^{\mathrm{dl}}\right)}^{2}E\left[|{\tilde{h}}_{g,k}^{H}\hat{H}{\left({\hat{H}}^{H}\hat{H}\right)}^{-1}e_{i}{|}^{2}\right]\\ \; & \overset{\left(c\right)}{=}{\left(q_{B,g}^{\mathrm{dl}}\right)}^{2}\left(\sum_{m=1}^{N_{B}}\kappa_{m,g,k}\right)+\sum_{i=1}^{G}\sum_{m=1}^{N_{B}}\left(\beta_{m,g,k}-\chi_{m,g,k}\right), \end{array}$$

where $\left(a\right)$ is because ${\tilde{h}}_{g,k}$ and ${\hat{h}}_{g,k}$ are independent, $\left(b\right)$ is obtained because that ${\hat{h}}_{g}$ and ${\hat{h}}_{i}$ are uncorrelated, $\left(c\right)$ results from the nature of expectation that $E\left[X_{1}X_{2}\right]=E\left[X_{1}\right]E\left[X_{2}\right]$ when $X_{1}$ and $X_{2}$ are independent.

Considering the term $\sum_{i=1}^{G}E\left[|g_{g,k}^{H}v_{i}^{\mathrm{ZF}}{|}^{2}\right]$, we have

(A8) $$\;\begin{array}{cc} \sum_{i=1}^{G}E\left[{\left|g_{g,k}^{H}v_{i}^{\mathrm{ZF}}\right|}^{2}\right]\; & =\sum_{i=1}^{G}E\left[|q_{S,i}^{\mathrm{dl}}g_{g,k}^{H}\hat{G}{\left({\hat{G}}^{H}\hat{G}\right)}^{-1}e_{i}{|}^{2}\right]\\ \; & \overset{\left(a\right)}{=}\sum_{i=1}^{G}{\left(q_{S,i}^{\mathrm{dl}}\right)}^{2}E\left[|c_{g,k}{\hat{g}}_{g}^{H}\hat{G}{\left({\hat{G}}^{H}\hat{G}\right)}^{-1}e_{i}{|}^{2}+|{\tilde{g}}_{g,k}^{H}\hat{G}{\left({\hat{G}}^{H}\hat{G}\right)}^{-1}e_{i}{|}^{2}\right]\\ \; & \overset{\left(b\right)}{=}{\left(q_{S,g}^{\mathrm{dl}}\right)}^{2}E\left[|c_{g,k}{\hat{g}}_{g}^{H}\hat{G}{\left({\hat{G}}^{H}\hat{G}\right)}^{-1}e_{g}{|}^{2}\right]+\sum_{i=1}^{G}{\left(q_{S,i}^{\mathrm{dl}}\right)}^{2}E\left[|{\tilde{g}}_{g,k}^{H}\hat{G}{\left({\hat{G}}^{H}\hat{G}\right)}^{-1}e_{i}{|}^{2}\right]\\ \; & \overset{\left(c\right)}{=}{\left(q_{S,g}^{\mathrm{dl}}c_{g,k}\right)}^{2}+\sum_{i=1}^{G}\left(\eta_{g,k}-\gamma_{g,k}\right), \end{array}$$

where $\left(a\right)$ is because ${\tilde{g}}_{g,k}$ and ${\hat{g}}_{g,k}$ are independent, $\left(b\right)$ is obtained because that ${\hat{g}}_{g}$ and ${\hat{g}}_{i}$ are uncorrelated, $\left(c\right)$ is obtained because of the nature of expectation that $E\left[X_{1}X_{2}\right]=E\left[X_{1}\right]E\left[X_{2}\right]$ for independent $X_{1}$ and $X_{2}$.

Substituting the above results into (17) yields the closed-form expression of the downlink achievable rate with ZF beamforming.
